# CRTC1 mediates preferential transcription at neuronal activity-regulated CRE/TATA promoters

**DOI:** 10.1038/s41598-017-18215-y

**Published:** 2017-12-21

**Authors:** Arnaldo Parra-Damas, Laura Rubió-Ferrarons, Jie Shen, Carlos A. Saura

**Affiliations:** 1 Institut de Neurociències, Department de Bioquímica i Biologia Molecular, Centro de Investigación Biomédica en Red Enfermedades Neurodegenerativas (CIBERNED), Universitat Autònoma de Barcelona, Barcelona, 08193 Spain; 2 Center for Neurologic Diseases, Department of Neurology, Brigham & Women’s Hospital, Harvard Medical School, Boston, MA 02115 USA

## Abstract

Gene expression mediated by the transcription factor cAMP-responsive element-binding protein (CREB) is essential for a wide range of brain processes. The transcriptional coactivartor CREB-regulated transcription coactivator-1 (CRTC1) is required for efficient induction of CREB target genes during neuronal activity. However, the mechanisms regulating induction of specific CREB/CRTC1-dependent genes during neuronal activity remain largely unclear. Here, we investigated the molecular mechanisms regulating activity-dependent gene transcription upon activation of the CREB/CRTC1 signaling pathway in neurons. Depolarization and cAMP signals induce preferential transcription of activity-dependent genes containing promoters with proximal CRE/TATA sequences, such as *c-fos*, *Dusp1*, *Nr4a1*, *Nr4a*2 and *Ptgs2*, but not genes with proximal CRE/TATA-less promoters (e.g. *Nr4a*3*, Presenilin-1* and *Presenilin-2*). Notably, biochemical and chromatin immunoprecipitation analyses reveal constitutive binding of CREB to target gene promoters in the absence of neuronal activity, whereas recruitment of CRTC1 to proximal CRE/TATA promoters depends on neuronal activity. Neuronal activity induces rapid CRTC1 dephosphorylation, nuclear translocation and binding to endogenous CREB. These results indicate that neuronal activity induces a preferential binding of CRTC1 to the transcriptional complex in CRE/TATA-containing promoters to engage activity-dependent transcription in neurons.

## Introduction

One of the most relevant features of the nervous system is its ability to sense, adapt and respond to environmental changes. These cellular responses occur in most cases through regulation of genetic programs that ensure appropriate changes in gene expression, a process mediated by transcription factors such as the cAMP-responsive element (CRE) binding protein (CREB)(^1^, for a review). By regulating hundreds of neuronal genes in response to a variety of extracellular stimuli, CREB mediates neuronal excitability, synaptic plasticity and long-term memory^2^. Synaptic transmission activates Ca^2+^ and cAMP signaling pathways leading to phosphorylation of CREB at Ser133, a required step for transcription of multiple genes^3^. CREB phosphorylation mediates binding of CREB to the histone acetyl-transferase (HAT) paralogues CREB binding protein (CBP) and p300, which leads to induction of transcriptional activity^4^. However, CREB phosphorylation is essential but not sufficient for gene transcription^5^, which suggests that multiple cellular mechanisms, including recruitment of coactivators act in concert with CREB phosphorylation to engage the transcriptional machinery.

CREB-regulated transcription coactivators (CRTCs) regulate multiple biological processes, including metabolism, cell transformation, memory and lifespan, and when disrupted cause age-related brain diseases^6,7^. The CRTC family comprises three members (CRTC1, CRTC2 and CRTC3) that are ubiquitously expressed, although CRTC1 is the main isoform expressed in neurons^8^. In resting conditions, CRTC1 is phosphorylated by the salt-induced kinase 1/2 (SIK1/2) promoting its interaction with 14-3-3 proteins and sequestration in the cytoplasm^9,10^. By contrast, CRTCs are activated by dephosphorylation and nuclear translocation in response to Ca^2+^ and cAMP signals. Synaptic activation increases cAMP levels and activates PKA, which phosphorylates and inhibits the SIK1/2 kinase, thereby preventing CRTC1 phosphorylation^11^. Simultaneously, Ca^2+^ influx through L-type voltage gated calcium channels activates the Ca^2+^-dependent phosphatase PP2B/calcineurin, which dephosphorylates CRTC1 inducing its translocation to the nucleus^11–14^. Once in the nucleus, CRTC1 binds to the bZIP domain of CREB, enhancing the interaction with the TATA box-binding protein (TBP)-associated factor TAF_II_130, a subunit of the transcription factor TFIID^9^. In neurons, CRTC1 dephosphorylation and nuclear translocation mediates activation of CREB target genes^11,15,16^, although how synaptic activity mediates CRTC1 recruitment and activation to specific gene promoters is still unclear.

The molecular mechanisms by which CRTC1 selectively binds and activates specific gene promoters in neurons are still largely unclear. The number of CRE sites, their distance to the transcription start site (TSS) and the presence of proximal TATA boxes within the promoter region, and the presence of coactivators seem to be the key determinants of transcriptional responsiveness to CREB in non-neuronal cells^17–19^. Notably, the specific contribution of each of these factors to activity-induced gene expression in neurons is unknown. Here, we report that neuronal activity triggers rapid and robust expression of CREB target genes containing both consensus CRE sites and TATA box sequences (CRE/TATA) in their proximal promoter region. On the contrary, genes with promoters containing consensus CRE sites lacking close TATA box sequences (CRE/TATA-less) are not efficiently induced by neuronal activity. Activity-dependent gene transcription in neurons involves CRTC1 dephosphorylation, binding to CREB complexes and recruitment to proximal CRE/TATA promoter regions. These results suggest that binding of CRTC1 to CREB/CBP into proximal CRE/TATA-containing promoter regions is a required step for robust activity-dependent gene transcription in neurons.

## Results

### Time-dependent activation of CREB-dependent transcription in neurons

**Figure 1:**
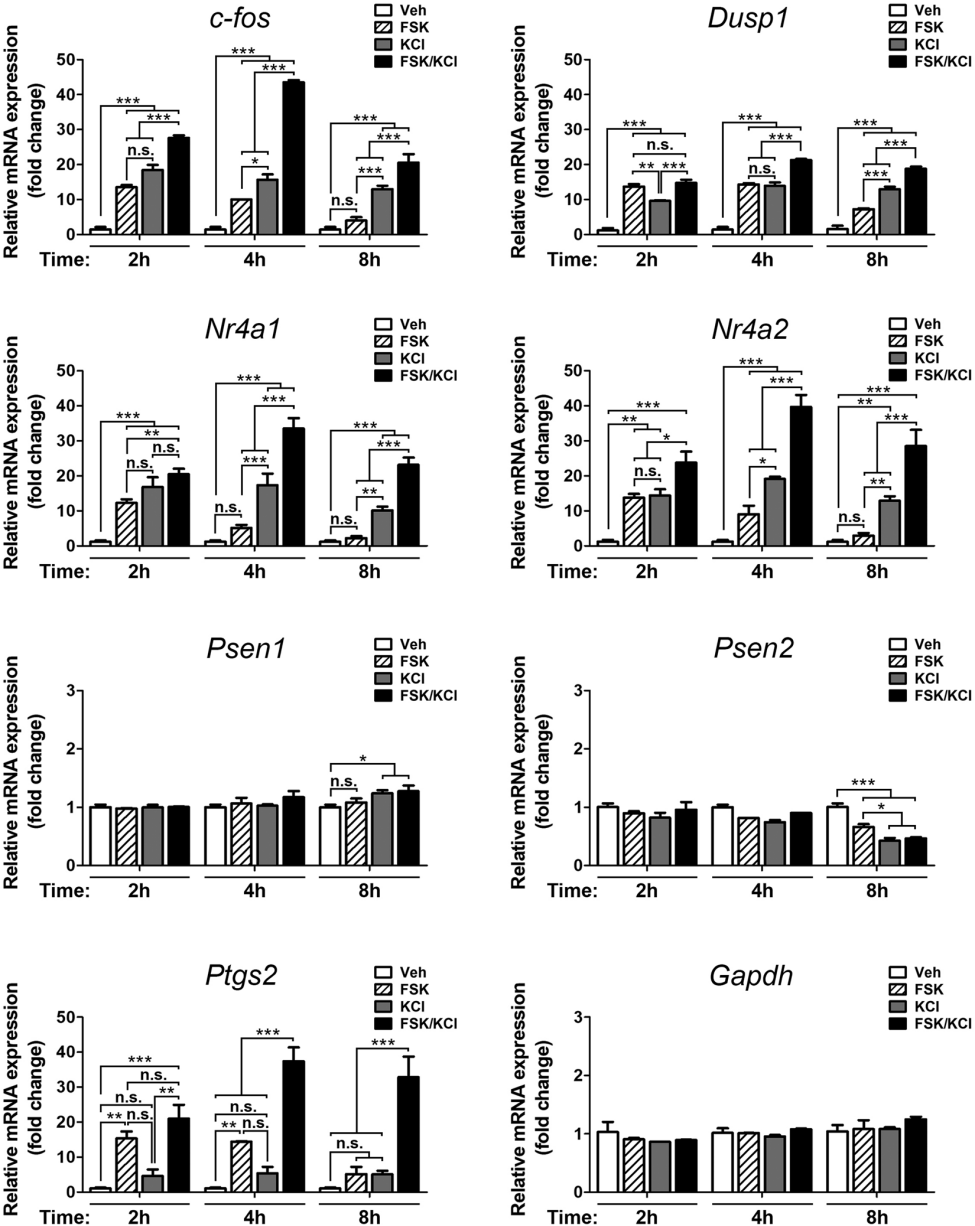
Neuronal activity induces CREB-dependent gene expression in primary cortical neurons. Quantitative real-time RT-PCR analysis of mRNA levels of CREB target genes in cultured cortical neurons (10 DIV). Levels of *c-fos*, *Dusp1*, *Nr4a1*, *Nr4a2* and *Ptgs2* mRNAs were differentially increased after KCl, FSK or FSK/KCl treatment in a time-dependent manner. On the contrary, *Gapdh* mRNA levels were not significantly different between vehicle- (Veh) and FSK/KCl-treated neurons at any time point, while *Psen1* and *Psen2* mRNA levels were significantly changed only after 8 hours of stimulation. Data represent fold change ± s.e.m relative to vehicle (Veh)-treated neurons from three independent experiments (n = 3). Gene expression levels were normalized to the geometric mean of *Ppia*, *Tbp* and *Gapdh*. N.s: non-significant. *P < 0.05, **P < 0.01, ***P < 0.001, compared to the indicated experimental group as determined by two-way ANOVA followed by Bonferroni test.

To study the mechanisms that regulate CREB-dependent transcription in neurons, we first examined the promoter regions and levels of genes containing canonical CRE sequences. CRE/TATA and CRE/TATA-less sites were defined based on the presence (CRE/TATA) or absence (CRE/TATA-less) of a TATA box within 300 bp downstream of the CRE site, according to the CREB Target Gene database [http://natural.salk.edu/CREB; ref.^18^], and regardless whether they are located proximal (−500 bp to +300 bp) or distal (>−500 bp) from the transcription start site (TSS). The number of CRE sites for the analized genes are: *c-fos* (6), *Dusp1* (2), *Nr4a1* (6), *Nr4a2* (6), *Psen1* (3), *Psen2* (2) and *Ptgs2* (2) (Table 1). Gene expression was evaluated by measuring mRNA levels with quantitative RT-PCR (qPCR) in cultured cortical neurons in basal and stimulated conditions. We used conditions mimicking neuronal activity by increasing the levels of intracellular Ca^2+^ and/or cAMP with depolarizing concentrations of KCl and the adenylate cyclase activator forskolin (FSK), respectively. Treatment of cortical neurons with FSK or KCl resulted in a differential time-dependent increase of mRNA levels of *c-fos* (~10–20 fold; treatment, F_(3,18)_ = 298.9, *P* < 0.0001; time, F_(2,18)_ = 45.48, *P* < 0.0001; interaction, F_(6,18)_ = 20.49, *P* < 0.0001), *Dusp1* (~5–15 fold; treatment, F_(3,18)_ = 324.2, *P* < 0.0001; time, F_(2,18)_ = 19.11, *P* < 0.0001; interaction, F_(6,18)_ = 14.32, *P* < 0.0001), *Nr4a1* (~5–10 fold; treatment, F_(3,18)_ = 154.3, *P* < 0.0001; time, F_(2,18)_ = 12.91, *P* = 0.0003; interaction, F_(6,18)_ = 8.10, *P* = 0.0002), *Nr4a2* (~5–15 fold; treatment, F_(3,18)_ = 99.40, *P* < 0.0001; time, F_(2,18)_ = 7.48, *P* = 0.004; interaction, F_(6,18)_ = 4.32, *P* = 0.007) and *Ptgs2* (~5–15; treatment, F_(3,18)_ = 68.02, *P* < 0.0001; time, F_(2,18)_ = 2.44, *P* = 0.1152; interaction, F_(6,18)_ = 3.55, *P* < 0.017), but not *Psen1* (~1–1.3 fold; treatment, F_(3,18)_ = 2.90, *P* = 0.064; time, F_(2,18)_ = 5.73, *P* = 0.0119; interaction, F_(6,18)_ = 1.27, *P* = 0.318) or *Psen2* (~1–0.5 fold treatment, F_(3,18)_ = 20.49, *P* < 0.0001; time, F_(2,18)_ = 29.61, *P* < 0.0001; interaction, F_(6,18)_ = 4.92, *P* < 0.004) (Fig. 1). As control, *Gapdh* mRNA was unchanged in all conditions (treatment, F_(3,18)_ = 0.40, *P* = 0.756; time, F_(2,18)_ = 3.11, *P* = 0.071; interaction, F_(6,18)_ = 0.4189, *P* = 0.856). Post hoc analyses revealed that FSK plus KCl induced in combination a significant additive or synergistic effects on expression of *c-fos* (~30–45 fold), *Dusp1* (~15–20 fold), *Nr4a1* (~20–30 fold), *Nr4a2* (~20–40 fold) and *Ptgs2* (~20–40) (Fig. 1). This result indicates a differential contribution of Ca^2+^ and cAMP signals on CREB-dependent transcription in cortical neurons.

**Table 1:** Consensus CRE sites in the promoter regions of CREB target genes in *Mus musculus*.

Symbol	Gene Name	Accession	Chrom	Strand	Position	CRE Sites	Total CRE sites	Proximal CRE (−500b to 300b)	Distal CRE (−3Kb to −500b)	Proximal CRE TATA
*cFos*	FBJ osteosarcoma oncogene	NM_010234	chr12	+	80146346	ht_−890 H_−349 HT_−301 ht_−138 HT_−75 H_175	**6**	**5**	**1**	**3**
*Dusp1*	dual specificity phosphatase 1	NM_013642	chr17	−	25199351	FT_−153 HT_−201	**2**	**2**	**0**	**2**
*Nr4a1*	nuclear receptor subfamily 4, group A, member 1	NM_010444	chr15	+	101707146	h_−2529 ht_−1402 H_−247 H_−227 HT_−83 HT_−54	**6**	**4**	**2**	**2**
*Nr4a2*	nuclear receptor subfamily 4, group A, member 2	NM_013613	chr2	−	57926892	H_−1596 H_−1576 H_−1480 FT_171 HT_256 HT_266	**6**	**3**	**3**	**3**
*Nr4a3*	nuclear receptor subfamily 4, group A, member 3	NM_015743	chr4	+	46911756	ht_−2698 ht_−1292	**2**	**0**	**2**	**0**
*Psen1*	presenilin 1	NM_008943	chr12	+	78349910	ht_−1839 ht_−1816 h_244	**3**	**1**	**2**	**0**
*Psen2*	presenilin 2	NM_011183	chr1	−	180982326	f_−4469 ht_−3526 ht_−3464	**3**	**0**	**3**	**0**
*Ptgs2*	prostaglandin-endoperoxide synthase 2	NM_011198	chr1	+	150628224	h_−457 ht_−78	**2**	**2**	**0**	**1**

Promoter elements according to the CREB Target Gene database (http://natural.salk.edu/CREB)^18^. Number of CRE sites in gene promoters classified according to the distance to the transcription starting site as follows: total, proximal (−500 bp to 300 bp), distal (−3 Kbp to −500 bp) and proximal CRE/TATA (with TATA boxes <300 bp). The position of each CRE relative to the TSS is indicated following the underlied dash. “FT_”: conserved full CRE site (TGACGTCA/TGACGTCA) containing a downstream TATA box at less than 300 bp;.“f_”: non-conserved full CRE site without TATA; “H_” or “HT_” mean a half CRE site (TGACG/CGTCA) conserved between species without (H) or with (HT) a TATA box less than 300 bp, respectively; “h_-” or “ht_-” mean a non-conserved half CRE site (TGACG/CGTCA) without (h) or with (ht) a TATA box less than 300 bp, respectively.

To test whether the proximity of CRE sites to the TSS and/or presence of TATA boxes in the promoter could affect gene expression, we examined the levels of *Psen1* and *Psen2*, which contain promoters with distal consensus CRE/TATA sites (*Psen1*: −1839 and −1816 bp; *Psen2*: −3526 and −3464 bp) and proximal CRE/TATA-less sites (*Psen1*: 244 bp) from the TSS in both mouse and human genes (Table 1 and Supplementary Table S1). Interestingly, treatment did not significantly affect levels of *Psen1* or *Psen2* at any stimulation time, except for a slight change at 8 h (Fig. 1). These results suggest that efficient activity-dependent transcription of CREB target genes in neurons depend on proximal CRE/TATA-containing promoter regions.

### Activity-dependent CRTC1 activation in neurons

**Figure 2:**
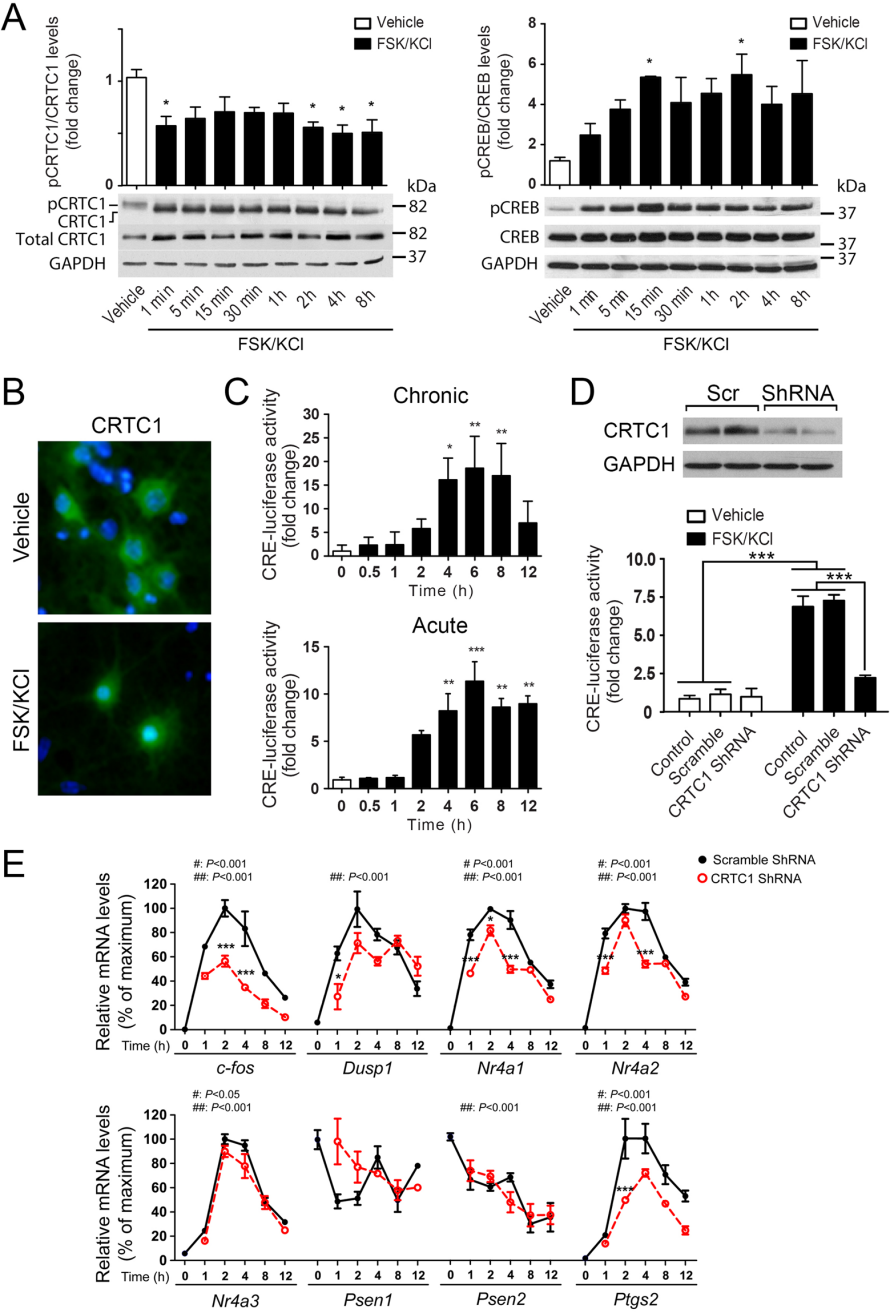
Activity-dependent CRTC1 activation in cultured neurons. (**A**) Activity-dependent changes on CRTC1 and CREB phosphorylation in cultured primary neurons. Western blot and quantitative analysis of pCRTC1, CRTC1, pCREB (Ser133) and CREB in 10 DIV cortical neurons treated with vehicle or FSK/KCl for 0–8 h. Values represent fold change ± s.e.m (n = 3 experiments). (**B**) Nuclear translocation of CRTC1 induced by neuronal activity. Representative immunofluorescence image of CRTC1 (green) and nuclear (Hoeschst 33324, blue) staining in neurons treated with vehicle or FSK/KCl for 15 min. (**C**) Time-dependent CRE-mediated transcription measured by a CRE-luciferase activity assay in primary neurons treated with FSK/KCl chronically (0–12 h) or acutely (1 h) and then washed and incubated for 0–12 h. Values represent fold change ± s.e.m of independent experiments (n = 3) performed in triplicate. (**D**) Western blot showing reduced CRTC1 levels in neurons treated with lentiviral *Crtc1* ShRNA (top panel). Reduced CRE-mediated transcriptional activity in *Crtc1* ShRNA-treated neurons after 4 h of treatment (bottom panel). Values represent fold change ± s.e.m (n = 3 independent transfections in duplicates). (**E**) CRTC1 regulates expression of CREB target genes in an activity-dependent manner. Real-time qRT-PCR analysis of mRNA levels normalized to *Gapdh* in neurons infected with scramble ShRNA (black dots) and *Crtc1* ShRNA (red dots). Data represent mean percentage ± s.e.m of three independent experiments performed by triplicate. “^#”^ and “^##”^represent statistical differences on time and treatment, respectively. *P < 0.05, ***P < 0.001, compared to scramble ShRNA-infected neurons in a specific time point. Statistical analysis was determined by two-way ANOVA followed by Bonferroni test.

We next studied the molecular mechanisms that regulate activity-dependent CREB transcription in neurons by focusing on CRTC1, a CREB transcriptional coactivator highly expressed in neurons of cortex and hippocampus^8,9^. FSK/KCl induced a significant time-dependent increase of CREB phosphorylation (Ser133) reaching a peak at 15 min (F_(8,26)_ = 2.4, *P* < 0.05; Fig. 2A). By contrast, chemical-induced neuronal activity induced a rapid (t_1/2_ ≈ 1 min) and persistent dephosphorylation of CRTC1 in cultured neurons (F_(8,26)_ = 2.9, *P* < 0.03), which is associated with translocation of CRTC1 from the cytosol to the nucleus (Fig. 2A,B).

To study the biological significance of CRTC1 dephosphorylation, we next measured CREB transcriptional activity by using a CRE-containing promoter luciferase assay. CRE-transcriptional activity was similarly induced in a time-dependent manner by chronic or acute neuronal activity (chronic, F_(7,22)_ = 8.5, *P* < 0.0003; acute, F_(7,23)_ = 14.0, *P* < 0.0001; Fig. 2C). A *Crtc1* shRNA that decreases *Crtc1* mRNA and protein levels (~75%) was able to reduce significantly activity-induced CREB transcriptional activity (group x treatment effect: F_(2,12)_ = 30.1, *P* < 0.0001; Fig. 2D **)** ^15^. In agreement with the above results, neuronal activity rapidly (t_1/2_ < 1 h) and significantly increased transcript levels of the canonical CREB target genes *c-fos* (fold change at 4 h: 40), *Dusp1* (10–12 fold), *Nr4a1* (40 fold), *Nr4a2* (50 fold) and *Ptgs2* (20 fold), whereas changes in *Nr4a3* (4 fold) and *Psen1/Psen2* (0.6 fold) were modest (Time effect for all genes: *P* < 0.001; Fig. 2E). *Crtc1* inactivation significantly decreased levels of *c-fos*, *Dusp1* (only significant at 1 h), *Nr4a1, Nr4a2* and *Ptgs2 (*Treatment effect: *P* < 0.001), but not those of *Nr4a3*, *Psen1* or *Psen2* (Fig. 2E). These results show rapid and robust activation of CRTC1-mediated transcription by neuronal activity in neurons.

### Activity-dependent recruitment of CRTC1 to target gene promoters

**Figure 3:**
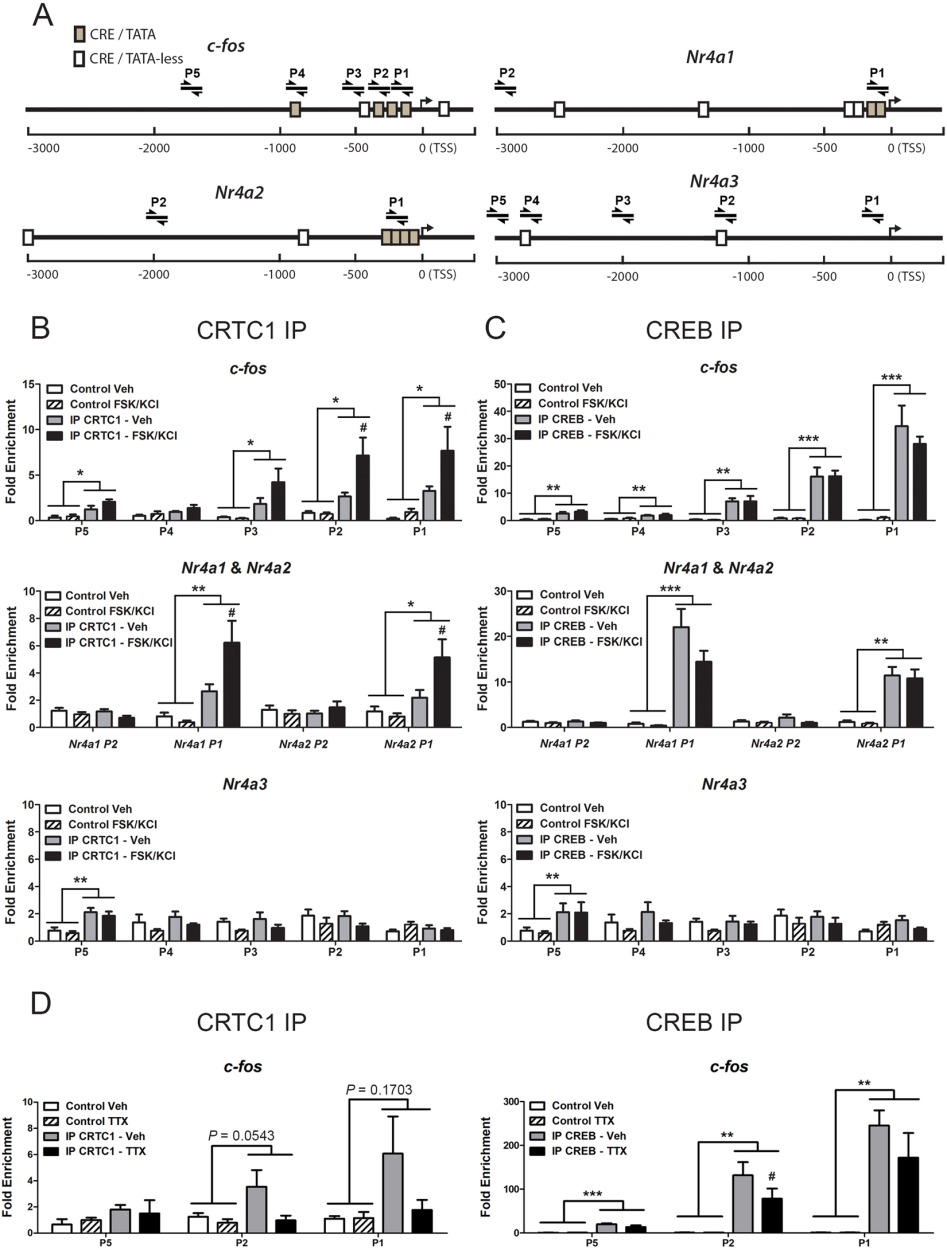
Differential recruitment of CRTC1 and CREB to target gene promoters upon neuronal activity. (**A**) Schematic representation of gene promoters of CREB target genes and position of amplified gene regions with specific primers (P1-P5) relative to CRE/TATA-less (white boxes) and CRE/TATA (grey boxes) sites relative to the transcription start site (TSS). (**B**,**C**) Quantitative chromatin immunoprecipitation (IP) analysis showing differential binding of CRTC1 (**B**) and CREB (**C**) to *c-fos*, *Nr4a1*, *2* and *3* gene promoter regions in primary cortical neurons treated with vehicle (Veh) or FSK/KCl for 15 min. The ratio between the immunoprecipitated chromatin obtained with beads (Control), anti-CRTC1 or anti-CREB antibodies or and the input chromatin is shown as fold enrichment of the amplified target regions over an irrelevant region in chromosome 4. (**D**) Chomatin IP analysis showing binding of CRTC1 and  CREB to the  CRE/TATA proximal promoter region of *c-fos* (P1-P2). CRTC1 binding to P1 and P2 regions is reduced but not significantly after TTX treatment (12 h). Data in (**B**–**D**) represent fold enrichment ± s.e.m of immunoprecipitation assays from several neuron cultures (n = 3–5). For each primer, *P < 0.05, **P < 0.01 and ***P < 0.001 represent statistical significance between groups as indicated using two-way ANOVA, whereas ^#^ *P* < 0.05 represent statistical significance between each group IP CRTC1- Veh and IP CRTC1-FSK/KCl in (**B**) and IP CREB-Veh and IP CREB-TTX in (**D**) using posthoc Bonferroni test.

The above results suggest that neuronal activity might differentially activate expression of genes containing CRE sites in their promoters. Gene sequence analyses revealed that the number of proximal CRE sequences (−500 bp to 300 bp of the TSS) containing TATA boxes (CRE/TATA) in the promoter regions differ substantially among the analyzed genes both in human and mouse genomes (Table 1, Supplementary Table S1). For the following analyzed mouse genes are indicated in parenthesis the number of proximal total CRE and CRE/TATA boxes: *c-fos* (5, 3), *Dusp1* (2,2), *Nr4a1* (4,2), *Nr4a2* (3,3), *Nr4a3* (0,0), *Psen1* (1,0), *Psen2* (0,0) and *Ptgs2* (2,1). We then explored the possibility that CREB and CRTC1 could bind differentially to the proximal promoter gene regions depending on the presence of CRE sites in close proximity to TATA boxes (CRE/TATA sites) by performing quantitative chromatin immunoprecipitation (ChiP-qPCR) analyses using CREB and CRTC1 antibodies and different set of primers amplifying distinct regions of CRE-containing promoters (Fig. 3A). In agreement with a previous report showing recruitment of CRTC1 into CREB target promoters^16^, neuronal activity induced a significant binding of CRTC1 to the proximal CRE/TATA promoter regions of *c-fos*, *Nr4a1* and *Nr4a2* (Fig. 3B). Notably, CREB binds constitutively to similar gene proximal promoter regions already in the absence neuronal stimulation (Fig. 3C). Conversely, we detected low binding of CREB and CRTC1 to a CRE/TATA-less site located distally of the TSS (−2698 bp) in the *Nr4a3* promoter region (Fig. 3B,C). To examine whether spontaneous neuronal activity was responsible for basal binding of CREB to gene promoters, we performed similar assays in the presence or absence of tetrodotoxin (TTX), a Na^+^ channel blocker that inhibits action potentials. In contrast to reduced binding of CRTC1 to the proximal *c-fos* promoter in the presence of TTX, basal binding of CREB to this promoter region was largely unaffected by TTX (Fig. 3D). These results suggest a preferential binding of CRTC1 to proximal CRE/TATA-containing target promoters in response to activity resulting in efficient CREB/CRTC1-dependent transcription in neurons.

**Figure 4:**
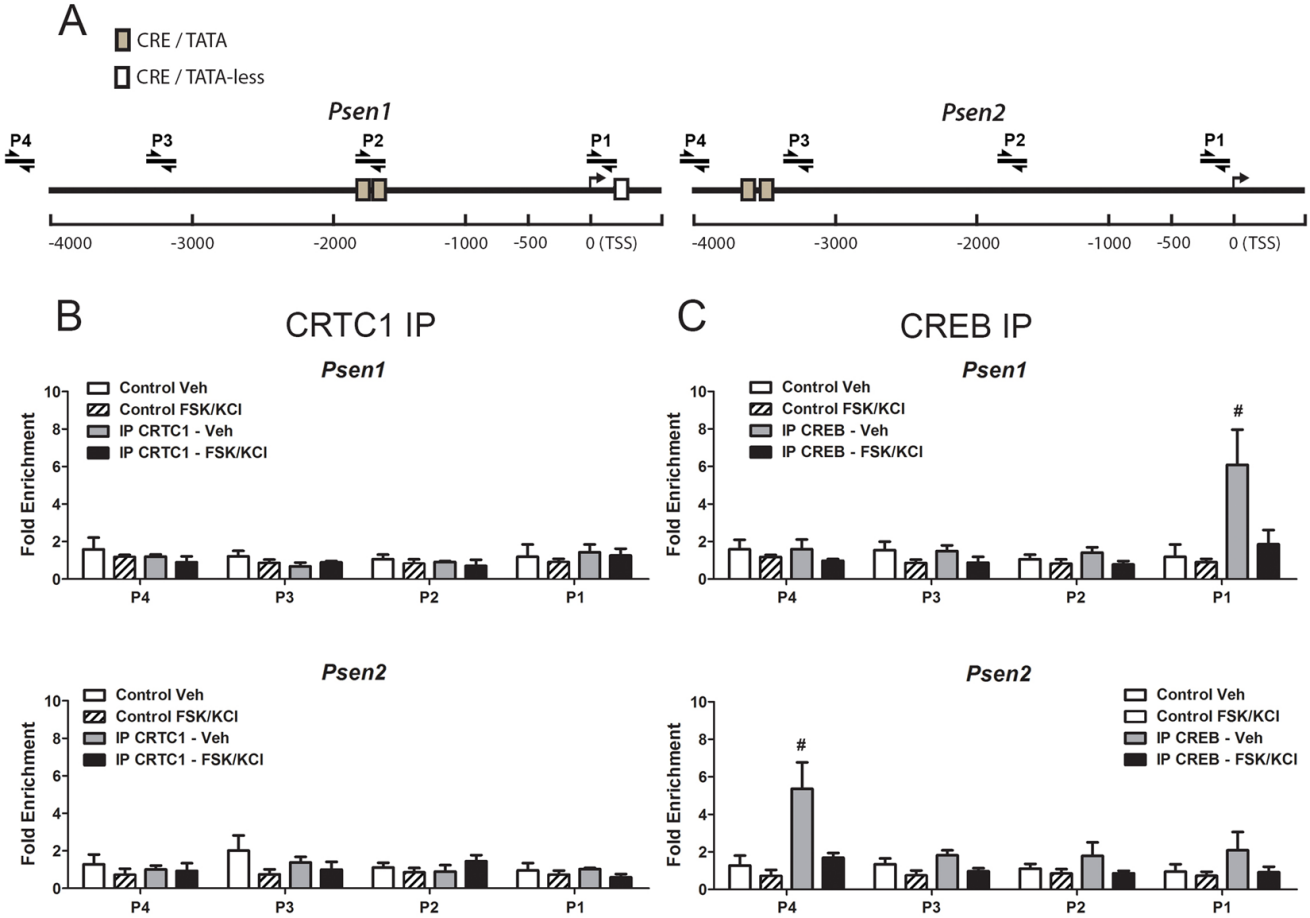
Deficient binding of CRTC1 and CREB to *Psen1* and *Psen2* promoters. (**A**) Schematic representation of *Psen1* and *Psen2* promoters and position of qPCR amplified gene regions with specific primers (P1-P4) relative to CRE/TATA-less (white boxes) and CRE/TATA (gray boxes) sites. TSS: transcription start site. (**B**,**C**) Quantitative ChIP analysis showing differential binding of CRTC1 (**B**) and CREB (**C**) to distinct *Psen1* and *Psen2* promoter regions using anti-CRTC1 (left) and anti-CREB (right) antibodies in cortical neurons treated with vehicle or FSK/KCl for 15 min. CRTC1 does not bind to *Psen1* and *Psen2* promoters. Data represent fold enrichment ± s.e.m of immunoprecipitation assays from independent cultures (n = 5). ^#^ *P* < 0.05 represents statistical significance of IP CREB Veh *vs* IP CREB FSK/KCl for P1 (*Psen1*) or P4 (*Psen2*), as determined by two-way ANOVA followed by Bonferroni test.

**Figure 5:**
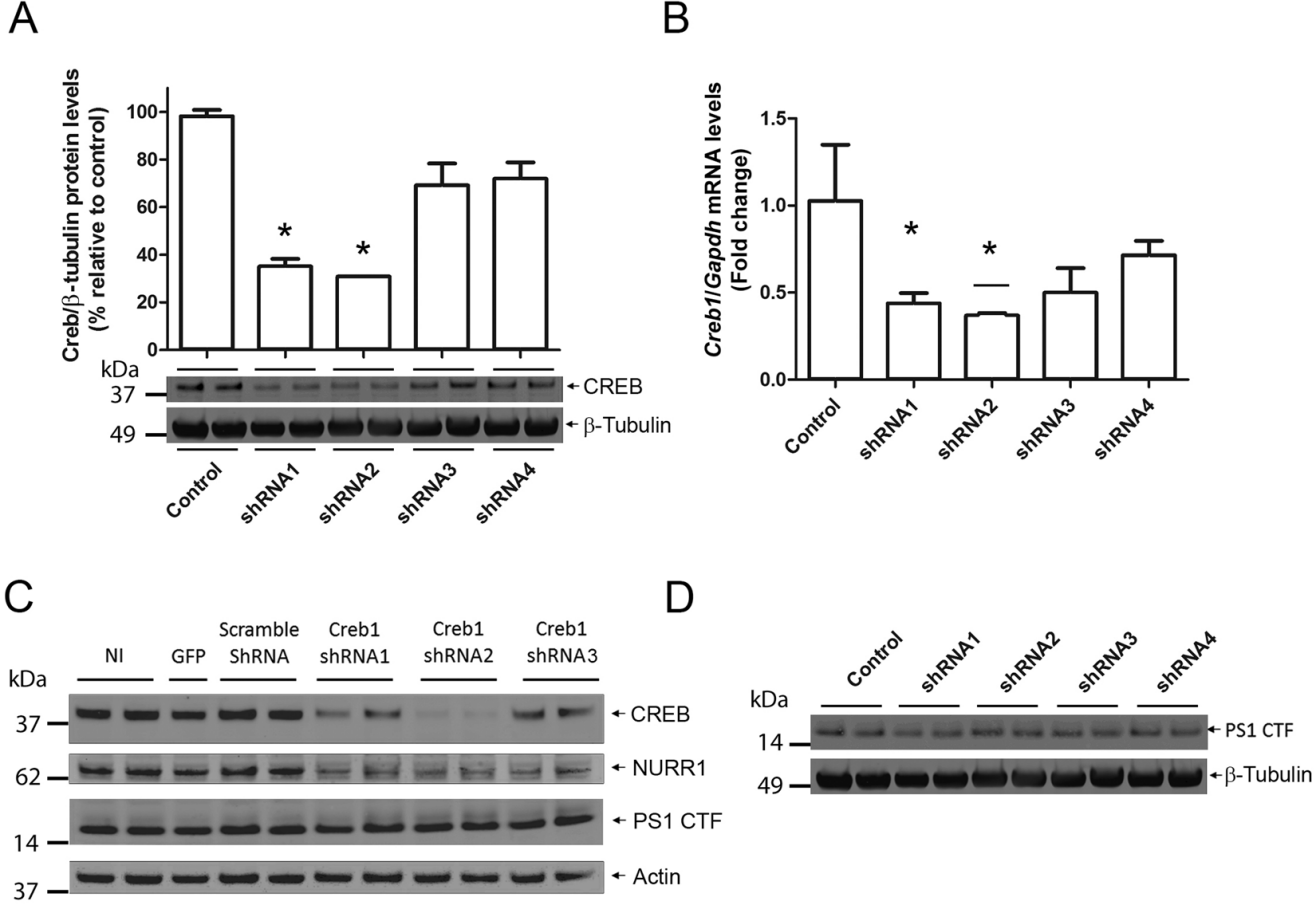
CREB silencing does not affect basal PS1 protein levels. (**A**,**B**) Genetic inactivation of CREB in primary cortical neurons (4 DIV) infected with lentiviral particles expressing scramble (control) or Creb1 shRNAs 1-4. Creb1 shRNA-1 and shRNA-2 reduced significantly total CREB protein (**A**) and mRNA (**B**) levels in primary cortical neurons. Data represent the mean fold change ± s.e.m relative to scramble shRNA (control) from independent experiments (n = 3). Levels of mRNA were normalized to *Gapdh*. *P < 0.05 as determined by one-way ANOVA followed by Dunnett’s multiple comparison test. (**C,D**) CREB silencing does not affect levels of PS1 C-terminal fragments (CTF) in human HEK-293T cells (**C**) and primary mouse cortical neurons (**D**) but efficiently reduces NR4A2 (NURR1) protein levels.

To evaluate further whether lack of proximal CRE/TATA sequences affects binding of CREB and CRTC1 to target gene promoters, we extended the ChIP-qPCR analysis to the promoter regions of *Psen1* and *Psen2*. CRTC1 does not bind to the promoter regions of *Psen1* and *Psen2* in basal or stimulated conditions (Fig. 4A,B). By contrast, we found a significant binding of CREB at a proximal CRE/TATA-less containing promoter region of *Psen1* (244 bp) in basal but not stimulated conditions (Fig. 4A,C **;** *P* < 0.05 Veh *vs* FSK/KCl). Similarly, CREB occupancy was significantly enriched close to the most distal CRE/TATA-less site of the *Psen2* promoter (−4412 bp) in basal but not stimulated conditions (*P* < 0.05 Veh *vs* FSK/KCl). Since CREB binds to the proximal promoter region of *Psen1* in basal conditions, it is possible that CREB could regulate *Psen1* gene expression independently of CRTC1. To test this idea, we developed four different ShRNAs (ShRNA 1–4) targeting the mouse CREB gene (*Creb1*). *Creb1* ShRNA1 and 2 significantly reduced *Creb1* protein (F_(4,14)_ = 76.86, *P* < 0.0001) and mRNA (F_(4,14)_ = 14.59, *P* < 0.001) in primary neurons (Fig. 5A,B). Notably, *Creb1* silencing reduced Nr4a2 (NURR1) protein levels but did not affect PS1 protein levels, as assayed with an antibody that recognize the PS1 C-terminal fragment (CTF), in human HEK-293T cells and cortical neurons (Fig. 5C,D). These results indicate that CREB does not regulate transcription of *Psen1*, which supports the idea that activity-dependent CRTC1/CREB binding to proximal CRE/TATA-containing promoter regions is required for optimal gene transcription.

### Neuronal activity induces binding of CRTC1 to the CREB transcriptional complex

**Figure 6:**
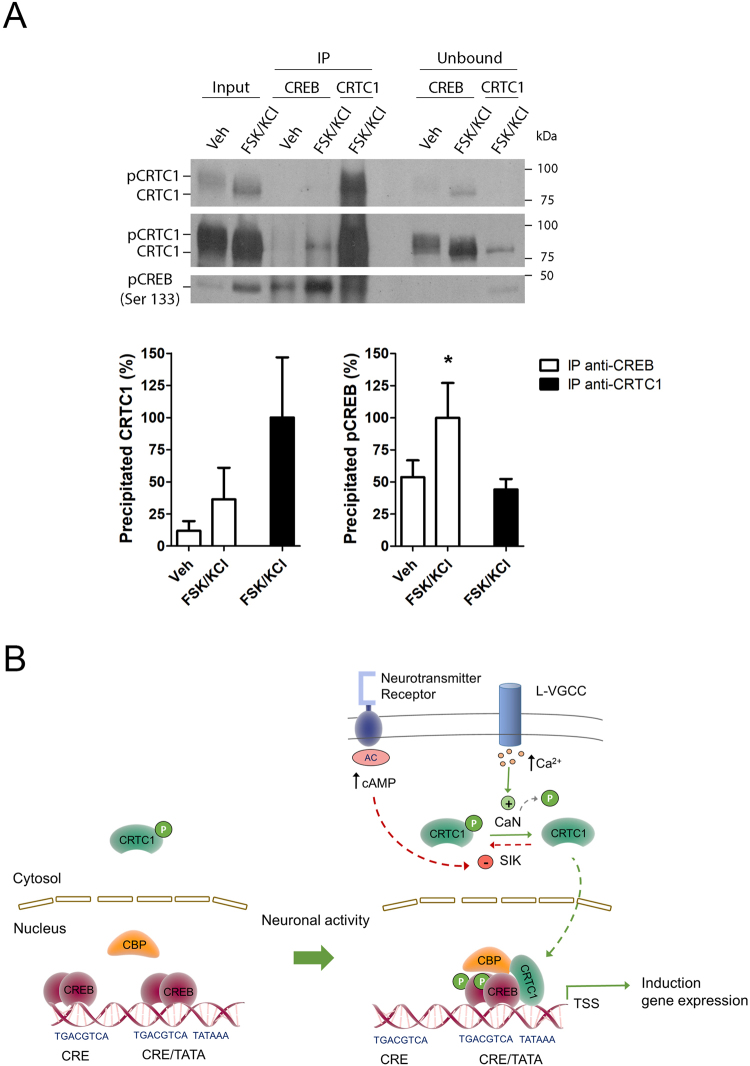
Activity-dependent binding of CRTC1 to CREB in neurons. (**A**) Coimmunoprecipitation experiments showing activity-dependent binding of endogenous CRTC1 and CREB in cultured cortical neurons. Representative immunoblots of CRTC1 (top: two different exposures) and pCREB (bottom) in the lysate (input), and immunoprecipitated (IP) and unbound fractions. Endogenous CRTC1 is immunoprecipitated with an anti-CREB antibody, and pCREB is efficiently immunoprecipitated with the anti-CRTC1 antibody in neurons treated with FSK/KCl. The graphs represent the percentage of immunoprecipitated CRTC1 and pCREB normalized to the maximum immunoprecipitation with their specific antibodies in FSK/KCl conditions, which is considered  100%. Data represent percentage of immunoprecipitation ± s.e.m of four independent experiments. *P < 0.05 by one-tailed t-test. (**B**) Proposed model of activity-dependent gene expression mediated by CRTC1 in neurons. In basal conditions, CREB binds constitutively to both CRE/TATA- and CRE/TATA-less-containing promoters of target genes. Synaptic activity activates neurotransmitter receptors and L-type voltage gated calcium channels (L-VGCC) elevating intracellular levels of the second messengers cAMP and Ca^2+^, which inhibits salt-induced kinase (SIK) and activates calcineurin/protein phosphatase 2B (CaN), respectively. This results in phosphorylation (P, in green) of CREB at Ser133 and CRTC1 dephosphorylation, nuclear translocation and preferential binding to CREB/CREB binding protein (CBP) complexes into CRE/TATA-containing promoters close to the transcription start site (TSS) to engage gene transcription.

It has been suggested that cooperative interactions between the CRTC isoform CRTC2 and CREB/CBP complexes regulate selectively gene transcription^12,20,21^. To test whether neuronal activity induces binding of endogenous CRTC1, the main isoform expressed in neurons, to the CREB transcriptional complex, we next performed coimmunoprecipitation experiments of endogenous CRTC1 and CREB in non-stimulated and stimulated cultured cortical neurons. FSK/KCl efficiently induced CREB phosphorylation and CRTC1 dephosphorylation in primary neurons (Fig. 6A). Immunoblotting confirmed successful immunoprecipitation of CRTC1 and phosphorylated CREB with their respective antibodies. Interestingly, immunoblotting revealed the presence of endogenous CRTC1 in CREB immunoprecipitates and CREB in CRTC1 immunoprecipitates in stimulated conditions (Fig. 6A). Indeed, in FSK/KCl conditions, the percentage of CREB (∼50%) that binds to CRTC1 is similar to the amount of CRTC1 (∼37%) bound to CREB. These results demonstrate that neuronal activity induces CRTC1 assembly into a CREB-containing transcriptional complex.

## Discussion

The transcription factor CREB activates the expression of hundred of genes in neurons^2^, but the molecular mechanisms regulating specific transcriptional programs in response to neuronal activity are still largely unclear. The elucidation of these transcriptional mechanisms may be important in cognitive disorders in which gene expression changes parallel synaptic plasticity and memory deficits^22^. This study reveals that whereas CREB binds constitutively to proximal CRE sequences of gene promoters, CRTC1 is preferentially recruited to target proximal promoter regions upon neuronal activity to engage activity-dependent gene transcription. Neuronal activity induces CRTC1/CREB-dependent transcription by a mechanism involving CRTC1 dephosphorylation, nuclear translocation and binding to CREB preferentially into proximal CRE/TATA-containing promoters. These results suggest a transcription model in which neuronal activity promotes cooperative interactions between CRTC1 and CREB/CBP into specific proximal target promoter regions to activate selectively expression of target genes in neurons (Fig. 6B).

The first interesting finding is that neuronal activity induces robust activation of CREB target genes containing proximal CRE/TATA consensus promoter sequences. Notably, whereas CREB binds constitutively to target gene promoters, robust activity-induced transcription is associated with increased recruitment of CRTC1 to the promoter proximal regions containing conserved CRE/TATA sequences. Basal recruitment of CREB to gene promoters is largely independent of neuronal activity since blocking spontaneous activity with TTX does not abolish its binding to the *c-fos* promoter. These results are consistent with genome-wide studies reporting constitutive binding of CREB to CRE-containing promoters, whereas depolarization increases binding of CREB and CBP at gene enhancers in neurons^18,23^. Indeed, CRTC1 was recently reported to bind to the proximal promoter regions of *c-fos* and *Bdnf IV* as well as to the distal (7 kb) half CRE site of a SARE enhancer element of *Arc* ^16,24^. In contrast to binding to proximal CRE/TATA-containing promoters of target genes (e.g *c-fos*, *Nr4a1* and *Nr4a2*), CRTC1 does not bind to CRE/TATA-less promoter regions of *Nr4a3*, *Psen1* and *Psen2*. This result is consistent with the requirement of proximal CRE/TATA sequences for efficient CREB-mediated transcription in non-neuronal cells^17^. The absence of CRTC1 recruitment to *Psen1* and *Psen2* promoters contrasts with basal binding of CREB to the proximal promoter regions of *Psen1*, a result suggesting the possibility that CREB could regulate *Psen1* transcription depending on the physiological conditions. For instance, neuronal activity reduces binding of CREB to the *Psen1* promoter while is recruited to other target gene promoters, a result resembling that observed when overexpressing CRTC1 *in vivo* ^24^. However, whereas neuronal activity does not affect *Psen1* mRNA levels in cortical neurons,  glutamate and BDNF activate *Psen1* expression in a CREB-dependent manner in neuroblastoma cells^25^. Notably, multiple consensus transcription binding sites are present in the promoter proximal region (−811 bp to +140 bp) of the human *PSEN1* gene including those for CREB, Lyf-1, CdxA, AML-1a, Nkx-2, Pbx-1, E47, MZF1, Sp1 and Elk-1^25^. Our biochemical studies showing that CREB inactivation does not affect *Psen1* levels in neurons reinforce the view that CREB is unlikely to be the main transcription factor regulating *Psen1* expression. Likewise, we also detected recruitment of CREB to a full CRE site located distally (−4412 bp) from the transcription start site of the *Psen2* promoter. It is relevant, however, that presenilins regulate CREB-mediated transcription in the adult brain likely through a mechanism involving the transcriptional coactivators CBP and CRTC1^26–28^. Future experiments will be necessary to elucidate the presenilin-regulated mechanisms of CREB-dependent transcription, and the role of proximal and distal CRE sites as regulatory elements (initiation, enhancers…) of the presenilin genes.

CREB-dependent transcription depends, at the molecular level, on CREB phosphorylation at Ser133^3,29,30^. However, although CREB phosphorylation is important it does not always correlate with activation of gene expression suggesting that phosphorylation kinetics and/or other transcriptional effectors regulate gene transcription^5,31,32^. Our results indicate that Ca^2+^ and cAMP signals have differential effects on expression of CREB target genes in neurons, whereas simultaneous Ca^2+^/cAMP signals result in additive or synergistic effects on gene expression. This differential effect on gene expression is unlikely due to differences on CREB phosphorylation. For instance, we found that Ca^2+^/cAMP signals induce gradual increase of CREB phosphorylation but rapid CRTC1 dephosphorylation in cortical neurons (see also ref.^33^). It is known that besides inducing similar levels of CREB phosphorylation, cAMP is more effective than stress signals in promoting CREB/CBP complex formation and gene transcription^34^. CBP recruitment to CREB is sufficient for CREB-mediated gene activation, whereas efficient transcriptional induction does not only requires CREB/CBP complex formation but also optimal cAMP signaling and histone acetylation^31,35,36^. Indeed, CRTCs potentiate CREB transcriptional activity through binding to CREB independently of CREB Ser133 phosphorylation^9,21^. Accordingly, our biochemical analysis showed that the Ca^2+^/cAMP-induced potentiation of gene transcription involves recruitment of CRTC1 to CREB/CBP complexes in target promoter regions. This result agrees with previous reports showing that Ca^2+^ and cAMP signals act synergistically on gene expression by activating CRTC1 or CRTC2 in neurons and non-neuronal cells, respectively^12,37^. Indeed, neuronal activity regulates CRTC1 nuclear translocation while cAMP modulates its persistence into the nucleus^11^. Conversely, Ca^2+^ and cAMP signals do not always cooperate to activate gene expression in response to neuronal activity^38^. The fact that genetic inactivation of CRTC1 blocks Ca^2+^/cAMP-mediated transcription strongly suggests that CRTC1 recruitment to CREB is essential for activity-induced CREB-dependent transcription in neurons. Importantly, neuronal activity and memory training activate preferentially CREB-dependent transcriptional programs^39,40^. In agreement, CRTC1 selectively regulates expression of CREB target genes involved in synaptic plasticity and memory, including among others *Bdnf*, *c-fos*, *Dusp1, Fgf1*, *Nr4a1* and *Nr4a2* ^16,24,36,37,41^.

In summary, this study shows that binding of CRTC1 to neuronal gene promoters depends on neuronal activity resulting in induction and/or maintenance of CREB-dependent transcription in neurons. Despite basal binding of CREB to gene promoters, efficient CREB-dependent transcription depends on activity-induced recruitment of CRTC1 to promoters containing proximal CRE/TATA elements. These results are consistent with the idea that the presence of consensus CRE/TATA sequences within the core promoter is required for efficient activation of CREB-dependent transcription mediated by interaction of CREB and the transcriptional complex in non-neuronal cells^9,17^. CRTC1 dephosphorylation and nuclear translocation are key molecular mechanisms triggering activity-dependent CREB target gene transcription in neurons^11,42^. Conversely, deficient dephosphorylation and nuclear translocation of CRTC1 cause transcriptional and memory deficits in experimental models of Alzheimer’s disease^24,43^. Future investigations on the mechanisms and effectors involved in CREB-dependent gene transcription may shed light on novel pathways and therapeutics in age-related cognitive disorders.

## Materials and Methods

### Generation of short hairpin RNA (shRNA)

*Crtc1* ShRNA lentivirus was generated as described^15^). *Creb1* shRNA sequences were designed using the siDESIGN Center tool (GE Healthcare Dharmacon) (http://dharmacon.gelifesciences.com/design-center/). The complete sequences of the oligos used to generate the *Creb1* shRNAs are as follows (target sequence is underlined).

Creb1_shRNA_1-Forward: 5′-**TCGTAGAAAGAAGAAAGAAT**TTCAAGAGAATTCTTTCTTCT TTCTACGTTTTTTGGAAC-3′

Creb1_shRNA_1-Reverse: 5′-TCGAGTTCCAAAAAACGTAGAAAGAAGAAAGAATTCTCTTG AAATTCTTTCTTCTTTCTACGA-3′

Creb1_shRNA_2-Forward: 5′-T**GAGCAATACAGCTGGCTAA**TTCAAGAGATTAGCCAGCTG TATTGCTCTTTTTTGGAAC-3′

Creb1_shRNA _2-Reverse: 5′-TCGAGTTCCAAAAAAGAGCAATACAGCTGGCTAATCTCTTG AATTAGCCAGCTGTATTGCTCA-3′

Creb1_shRNA_3-Forward: 5′-T**ACTGATGGACAGCAGA**TTCTATTCAAGAGATAGAATCTG CTGTCCATCAGTTTTTTTGGAAC-3′

Creb1_shRNA_3-Reverse: 5′-TCGAGTTCCAAAAAAACTGATGGACAGCAGATTCTATCTCTT GAATAGAATCTGCTGTCCATCAGTA

Creb1_shRNA_4-Forward: 5′-T**CAGCAGCTCATGCAACATCAT**TTCAAGAGAATGATGTTG CATGAGCTGCTGTTTTTTGGAAC-3′

Creb1_shRNA_4-Reverse: 5′-TCGAGTTCCAAAAAACAGCAGCTCATGCAACATCATTCTCTT GAAATGATGTTGCATGAGCTGCTGA-3′.

Oligos were annealed and cloned into the HpaI-XhoI sites of the lentiviral vector pLLX, downstream of the U6 promoter. Lentiviral particles were generated by co-transfecting HEK293T cells with pLLX-*Creb1* or scramble or pLVTHM-*Crtc1* or scramble shRNAs with psPAX2 (packaging), and pM2G (envelope) vectors using X-tremeGENE transfection reagent (Roche) as described^15^. Lentivirus-containing medium was collected 48 h later and lentivirus were purified and stored at −80 °C.

### Primary neuronal culture

Mouse embryonic brains (E15.5; C57BL/6/129 background) were dissected and cortices were digested in cold Hank’s Balanced Salt Solution (HBSS) containing 0.025% trypsin-EDTA (Thermo Fisher Scientific) and DNAse (Sigma). Trypsin activity was inhibited by adding 20% FBS and the tissue was dissociated in HBSS containing DNAse and MgSO_4_. Neurons were plated in poly-D-lysine (PDL) coated dishes at a density of 375 cells/mm^2^ using DMEM containing 5% FBS penicillin-streptomycin (25 U/ml penicillin, 25 μg/ml streptomycin, Thermo Fisher Scientific) and glutamine (1 mM, Thermo Fisher Scientific). Two hours after seeding, DMEM medium was replaced by Neurobasal medium containing B27 supplement (Thermo Fisher Scientific), penicillin-streptomycin (25 U/ml penicillin, 25 μg/ml streptomycin, Thermo Fisher Scientific) and glutamine (1 mM, Thermo Fisher Scientific). For gene inactivation, 4 DIV neurons were infected with *Crtc1* or *Creb1* ShRNA lentiviral vectors (1–2 transducing units/cell). Neuronal activity was induced by treating 7–10 days *in vitro* (DIV) neurons with 10 μM forskolin (FSK), 55 mM potassium chloride (KCl) or both for 0, 2, 4 and 8 hr as indicated^15^. In some experiments, neuronal activity was blocked by adding 1 μM tetrodotoxin (TTX, Tocris) at 11 DIV.

### Gene expression analysis

Primary neurons were infected at 4 DIV with scramble or *Crtc1* and *Creb1* ShRNAs lentiviral vectors (1–2 transducing units/cell) and treated with vehicle or KCl (30 mM) plus forskolin (20 μM; Sigma) for 0–8 h at 12 DIV. For luciferase assays, 7–15 DIV neurons in 24-well dishes were transfected using LipofectAMINE 2000 (Thermo Fisher Scientific) for 24 h with pCRE-luc reporter vector (0.5 μg; Stratagene), containing 4xCRE sequences and a TATA-box element, and pRL-TK *Renilla luciferase* plasmid (0.25 μg; Promega), which encodes *Renilla* luciferase gene driven by HSV-TK promoter in a pGL4.74 vector (0.25 μg; Promega). Neurons were treated with vehicle or FSK/KCl chronically (0–12 h) or acutely (1 h) and then washed and incubated for 0–12 h. Samples were analyzed by triplicate in at least three independent transfections with the dual-luciferase activity assay (Promega) in a Synergy HT luminometer (Bio-Tek) as described^15^.

For quantification of mRNA, RNA from cultured neurons or hippocampal tissue was purified using the PureLink RNA Mini Kit (Thermo Fisher Scientific). RNA integrity number (RIN) was measured using the Agilent 2100 bioanalyser (Agilent Technologies). RNA (1 μg; RIN >8.0) was reverse-transcribed in 50 μl of a reaction mix containing 1 μM of Oligo (dT) primers, 1 μM random hexamers, 0.5 mM dNTP, 0.45 mM DTT, RNAseOut (10 units) and SuperScript^TM^ II reverse transcriptase (Thermo Fisher Scientific) at 25 °C for 10 min, 42 °C for 60 min and 72 °C for 10 min. Quantitative real time RT-PCR (qRT-PCR) was performed in duplicate in at least 3–5 samples using an Applied Biosystems 7500 Fast Real-Time PCR system (Thermo Fisher Scientific). Data analysis was performed by the comparative ΔCt method using the Ct values and the average value of PCR efficiencies obtained from LinRegPCR software^44^. Gene expression was normalized to *Gapdh* for cultured neurons or the geometric mean of *Gapdh*, hypoxanthine guanine phosphoribosyl transferase (*Hprt*) and peptidylprolyl isomerase A (*Ppia*) for brain samples using the NormFinder, BestKeeper and geNorm algorithms^45^.

### Biochemical analysis

Neurons were lysed in cold lysis buffer (50 mM Tris-HCl, pH 7.4, 150 mM NaCl, 2 mM EDTA, 0.5% Triton X-100, 1% NP-40, 0.1% SDS, 1 mM Na_3_VO_4_, 50 mM NaF, 1 mM PMSF) containing protease and phosphatase inhibitors (Roche España, Barcelona, Spain). Proteins were quantified with the BCA protein assay kit (Thermo Fisher Scientific), resolved on 8–14% SDS-polyacrylamide gel electrophoresis (PAGE) and transferred to PVDF membranes before blotting with the following antibodies: rabbit anti-CRTC1 (1:10,000), CREB (1:250) and phosphorylated CREB (Ser133) (1:1,000; Cell Signaling, Danvers, Massachusetts), NURR1 (Ab41917; 1:1,000), β-actin (Ab8227; 1:10,000) and anti-GAPDH (1:5,000; Abcam, Cambridge,UK) and PS1 Loop (1:2,000; AB5308; Merck-Millipore, Darmstadt, Germany) or β-tubulin (1:20,000; T7816; Sigma-Aldrich/Merck). We used distinct percentages of acrylamide in the gels for separation of phosphorylated/dephosphorylated CRTC1 (8%; upper membrane in Fig. 2A) and total CRTC1 (12.5%; middle membrane in Fig. 2A). For coimmunoprecipitation assays, cortical neurons (12 DIV) were crosslinked with 1% formaldehyde in growing medium and lysed by sonication in ice-cold buffer (50 mM Tris-HCl pH8, 1% Triton X-100, 100 mM NaCl, 1 mM MgCl_2_, 1 mM PMSF and protease and phosphatase inhibitors). Protein (2 mg) was immunoprecipitated overnight at 4 °C with or without antibodies against CREB or CRTC1 (Cell Signaling) followed by incubation with Dynabeads protein G (Invitrogen). The beads were spun to obtain a supernatant (unbound fraction) and a pellet, which was washed in ice-cold buffer (x3) and eluted with 2x SDS sample buffer (immunoprecipitated fraction). Quantitative analysis of immunoblots, normalized to control loading protein, was performed by densitometry using ImageJ (NIH, Bethesda, MD). Original blots scans are shown in Supplementary Information.

### ChIP-qPCR analysis

Chromatin immunoprecipitation (ChIP) was performed essentially as described^24^. Briefly, 12 DIV cortical neurons were treated with vehicle or FSK (20 μM) and KCl (30 mM) for 15 min, or pretreated with tetrodotoxin (TTX; 1 μM) for 12 h. Cells were crosslinked with 1% formaldehyde, lysed in ChIP buffer (50 mM Tris-HCl pH 8.1, 100 mM NaCl, 5 mM EDTA, 1% SDS, 0,1% Na deoxycholate and protease/phosphatase inhibitors) and chromatin was sheared between 200 and 500 bp by sonication using a BioruptorPlus (Diagenode, Seraing, Belgium). Fragmented chromatin was analyzed using the High Sensitivity DNA Kit (Agilent Technologies). Immunoprecipitation (2.5 μg DNA) was performed overnight in diluted ChIP buffer (0.1% SDS, 1,1% Triton X-100) with or without anti-CRTC1 or CREB antibodies (Cell Signaling). Input and immunoprecipitated DNA were decrosslinked and amplified by real-time qPCR using specific primers, and the fold enrichment of the amplified target regions was calculated over an irrelevant region in the chromosome 4.

### Statistical analysis

Statistical analysis was performed using t-test, one-way or two-way analysis of variance (ANOVA) for multiple comparisons followed by Bonferroni or Dunnett’s tests using GraphPad Prism software. Differences with *P* < 0.05 were considered significant.

### Ethical experimental statement

This study was performed in accordance with the experimental European Union guidelines and regulations (2010/63/EU). Experimental protocols and experiments involving vertebrate animals were conducted in accordance with the ethical protocol approved by the Animal and Human Ethical Committee (CEEAH) of the Universitat Autònoma de Barcelona (protocol number: CEEAH 2896) and the local Governmental Ethical Committee of the Generalitat de Catalunya (protocol number: DMAH 8787).

### Data availability statement

All data generated during this study are included in this published article or are available from the corresponding author upon reasonable request.

## Electronic supplementary material

Supplementary Information
