# Supplementary material for: CRTC1 mediates preferential transcription at neuronal activity-regulated CRE/TATA promoters
Source: Sci Rep. 2017 Dec 21;7:18004. doi: 10.1038/s41598-017-18215-y (PMC5740062; doi:10.1038/s41598-017-18215-y)

Suplementary Information

CRTC1 mediates preferential transcription at neuronal activity-regulated CRE/TATA promoters

Arnaldo Parra-Damas, Laura Rubió-Ferrarons, Jie Shen and Carlos A. Saura

Supplementary Table 1. Consensus CRE sites in the promoter regions of CREB target genes in *H. Sapiens*

Symbol	Gene Name	Accession	Chrom	Strand	Position	CRE Sites	Total CRE sites	Proximal CRE (-500b to 300b)	Distal CRE (-3Kb to -500b)	Proximal CRE TATA
FOS	v-fos FBJ murine osteosarcoma viral oncogene homolog	NM_005252	chr14	+	73735571	HT_-343<br>HT_-296<br>HT_-63<br>H_190	**4**	**4**	**0**	**3**
DUSP1	dual specificity phosphatase 1	NM_004417	chr5	-	172179126	h_97<br>ht_-102<br>FT_-113<br>HT_-163<br>ht_-1239	**5**	**4**	**1**	**3**
NR4A1	nuclear receptor subfamily 4, group A, member 1	NM_002135	chr12	+	50731457	ht_-1234<br>ht_-683<br>HT_-197<br>HT_-177<br>HT_-34<br>HT_-5	**6**	**4**	**2**	**4**
NR4A2	nuclear receptor subfamily 4, group A, member 2	NM_006186	chr2	-	157391745	HT_-71<br>HT_-81<br>FT_-163<br>h_-493<br>H_-1936<br>H_-2033<br>H_-2053	**7**	**4**	**3**	**3**
PSEN1	presenilin 1 Transcript variant 1	NM_000021	chr14	+	71593219	h_-166<br>h_-32	**2**	**2**	**0**	**0**
PSEN1	presenilin 1 Transcript variant 2	NM_007318	chr14	+	71593565	h_-512<br>h_-378	**2**	**1**	**1**	**0**
PSEN2	presenilin 2 (isoform 1)	NM_000447	chr1	+	224033590	-	**0**	**-**	**-**	**-**
PSEN2	presenilin 2 (isoform 2)	NM_012486	chr1	+	224033590	-	**0**	**-**	**-**	**-**
PTGS2	prostaglandin-endoperoxide synthase 2	NM_000963	chr1	-	183888548	h_29<br>ht_-53<br>ht_-564	**3**	**2**	**1**	**1**

Promoter elements according to the CREB Target Gene database (http://natural.salk.edu/CREB) [18]. Number of CRE sites in gene promoters classified according to the distance to the transcription starting site as follows: total, proximal (-500 bp to 300 bp), distal (-3Kbp to -500bp) and proximal CRE/TATA (with TATA boxes < 300bp). The position of each CRE relative to the TSS is indicated following the underlied dash."FT_": conserved full CRE site (TGACGTCA/TGACGTCA) containing a downstream TATA box at less than 300 bp; "H_" or "HT_" mean a half CRE site (TGACG/CGTCA) conserved between species without (H) or with (HT) a TATA box less than 300 bp, respectively; "h_-" or "ht_-" mean a non-conserved half CRE site (TGACG/CGTCA) without (h) or with (ht) a TATA box less than 300 bp, respectively.

Original scans of Western blots

Figure 2

Figure 2A left panel: two different exposures of the same membrane are shown

pCRTC1 (upper band detected mainly in Vehicle treated neurons) and dephospho-CRTC1 (prominent lower band detected mainly at 1-8h):

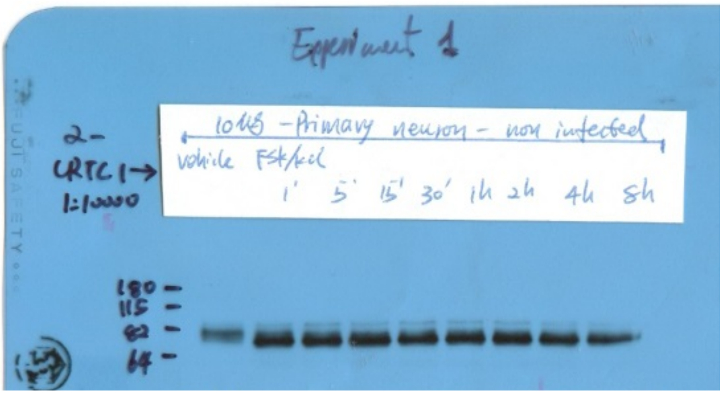

GAPDH:

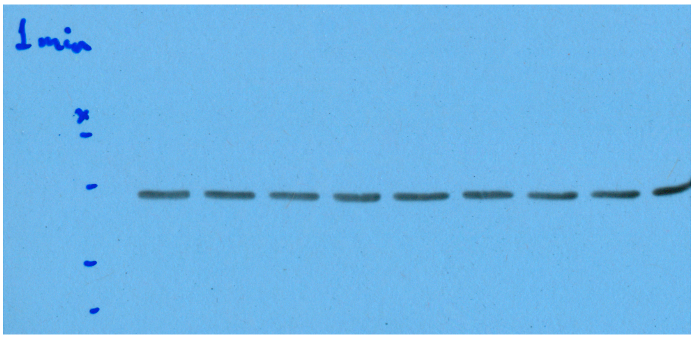

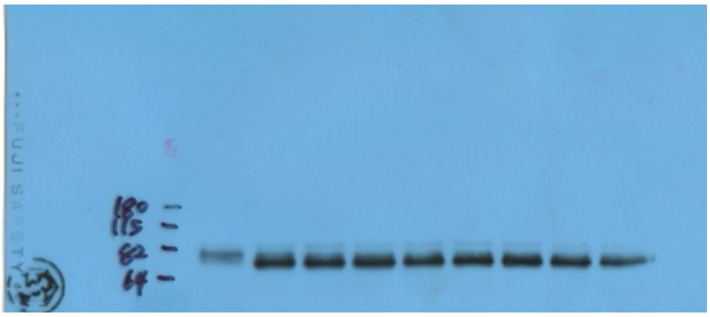

Figure 2A right panel:

pCREB (Ser133): Two different exposures are shown

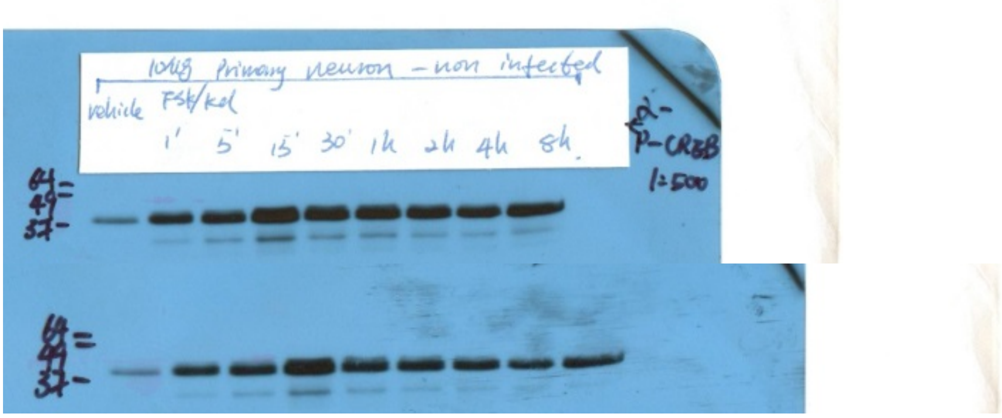

CREB:

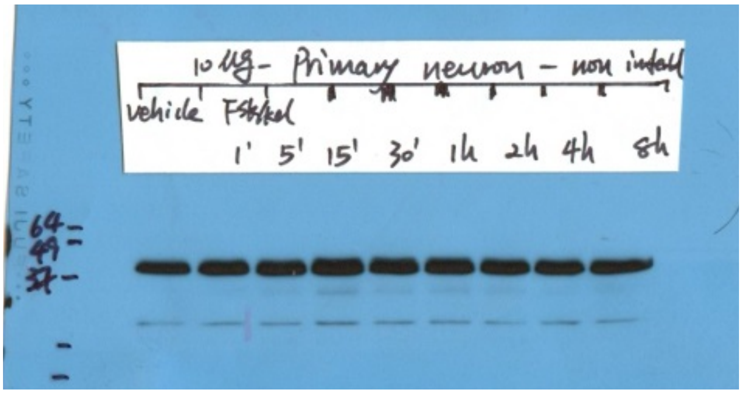

GAPDH:

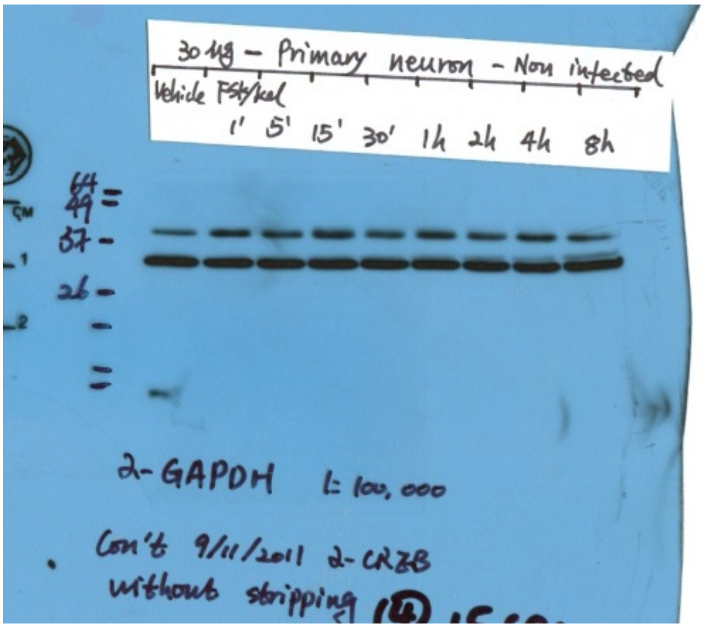

<u>Figure 2D</u>
CRTC1:

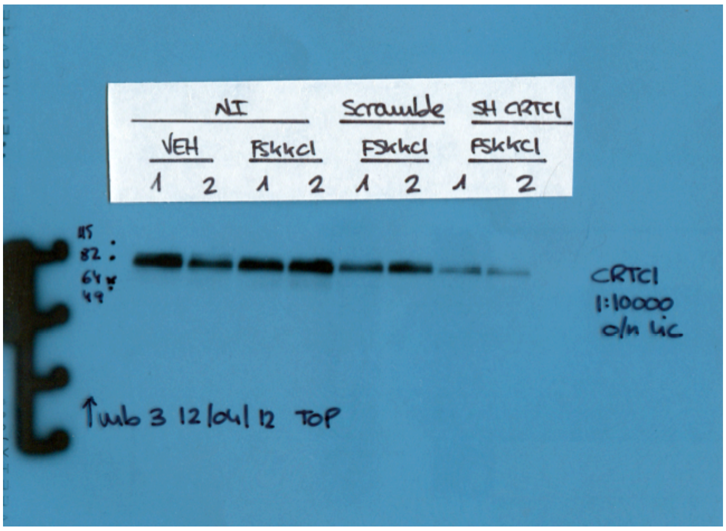

GAPDH:

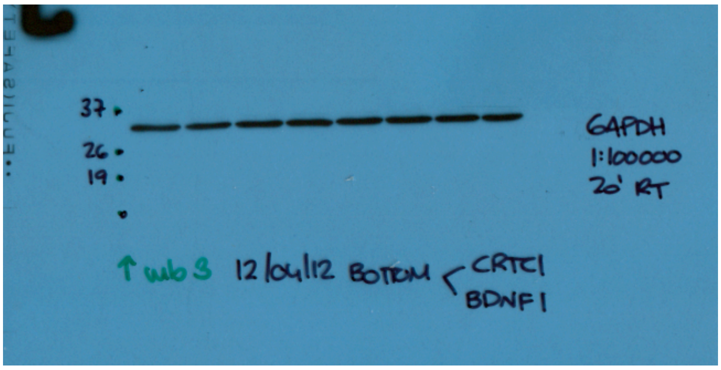

Figure 5

Figure 5A: Primary cortical neurons

CREB (Upper band):

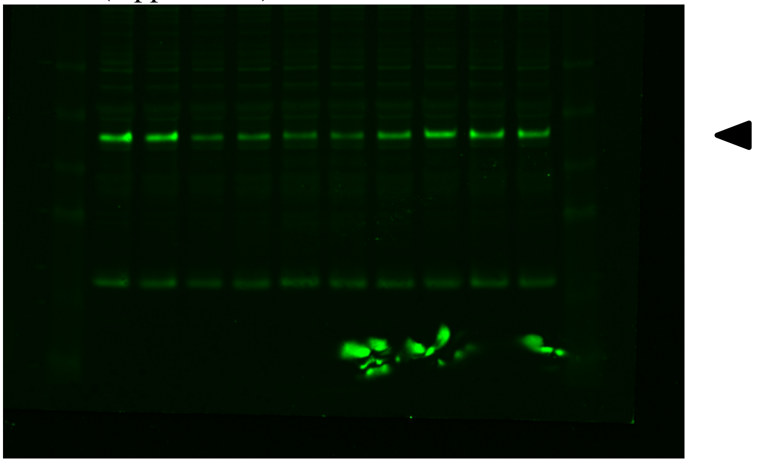

β-tubulin:

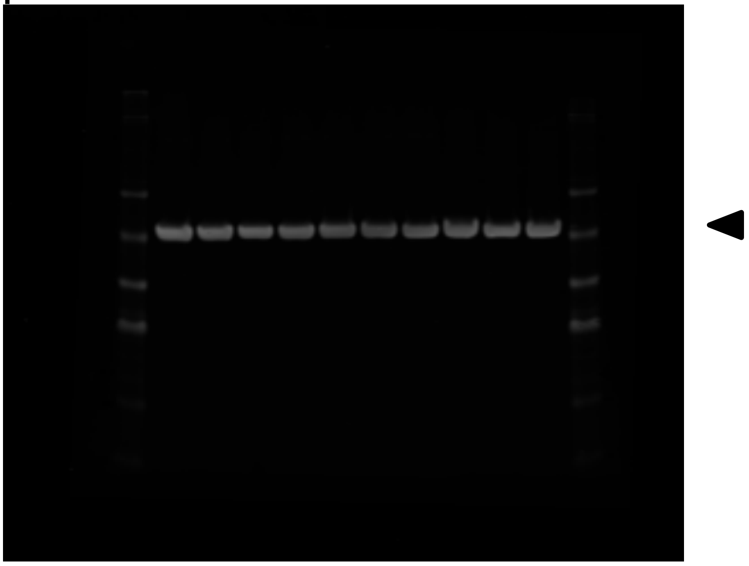

Figure 5C: HEK293 cells

Sequential blotting of the same membrane with the following antibodies:

CREB:

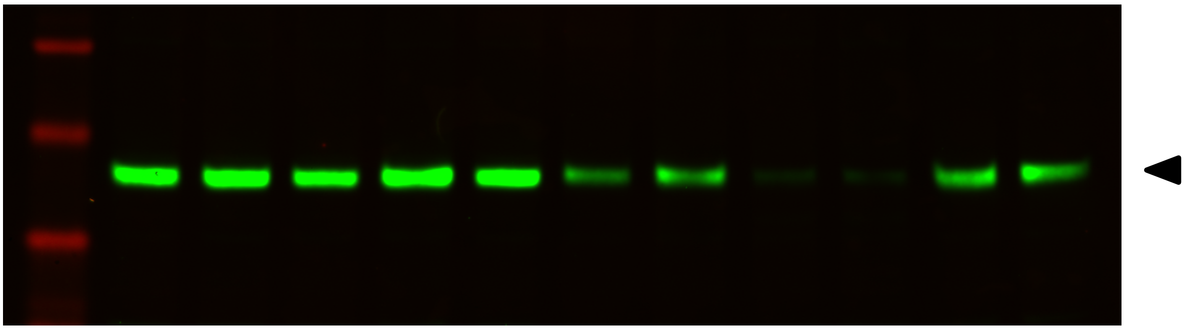

NURR1 (upper prominent band):

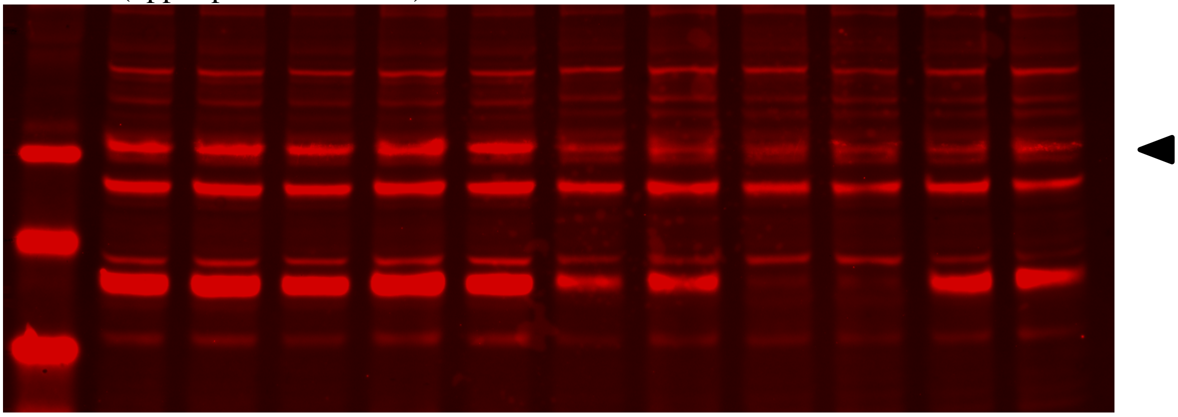

PS1 CTF (bottom band):

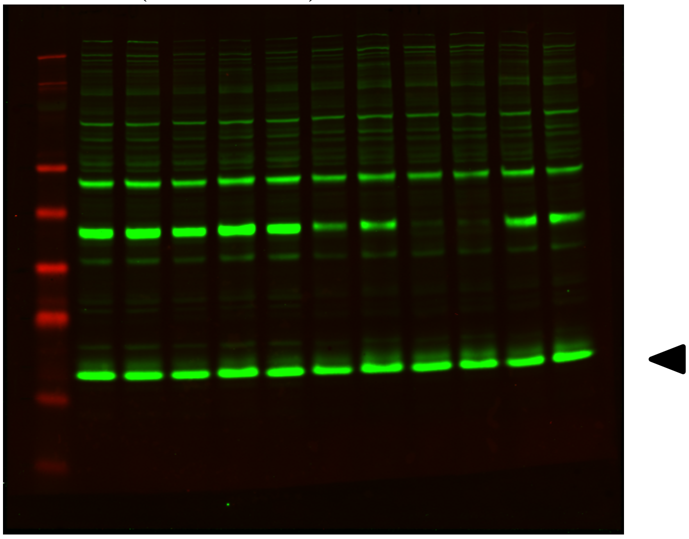

<u>Figure 5D</u>:
Same membrane than Figure 5A.
PS1 CTF (PS1 loop antibody; lower band)

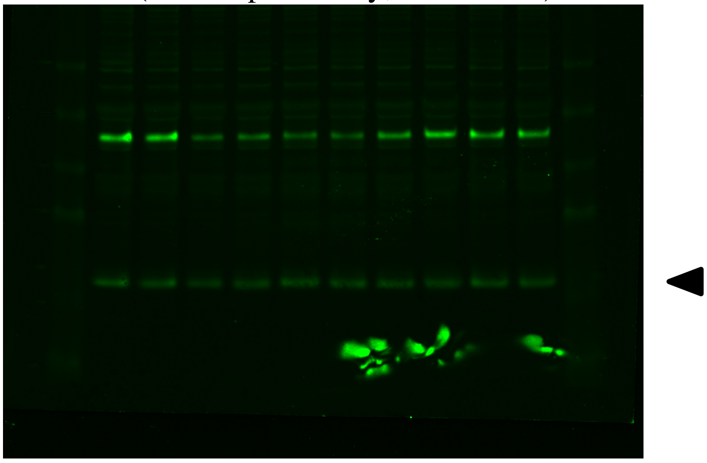

β-tubulin:

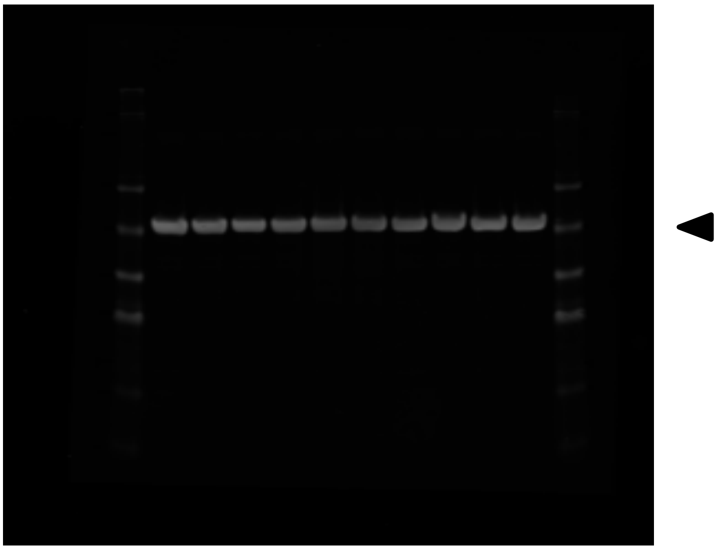

Figure 6

<u>Figure 6A</u>:

pCRTC1/CRTC1 : top (long exposure) and middle (short exposure) membranes and **phosphorylated Ser133 CREB** (pCREB; bottom membrane)

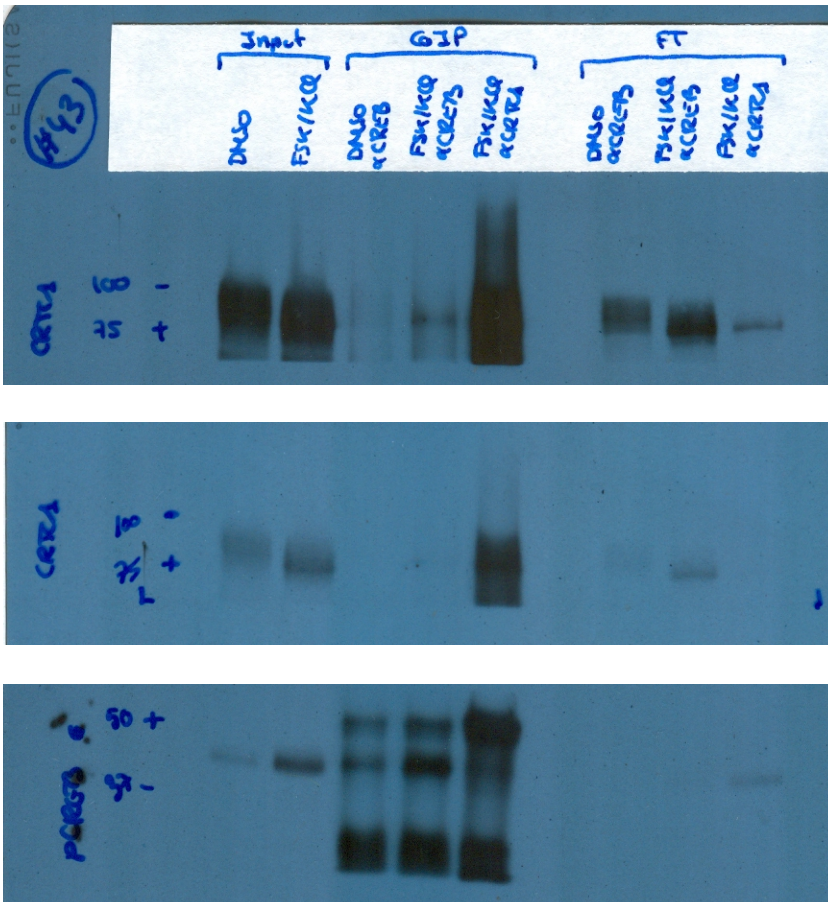

Supplement: Supplementary file 1 — Supplementary Information [file 41598_2017_18215_MOESM1_ESM.pdf]
